# Supplementary material for: The Systems Biology Research Tool: evolvable open-source software
Source: BMC Syst Biol. 2008 Jun 29;2:55. doi: 10.1186/1752-0509-2-55 (PMC2446383; doi:10.1186/1752-0509-2-55)
Supplement: Additional file 1 — SBRT Archive. An archive of the current version of the Systems Biology Research Tool. [file 1752-0509-2-55-S1.zip › sbrt-1.4.0/doc/developers_guide/api/sbrt/shell/class-use/ApplicationException.html]

Uses of Class sbrt.shell.ApplicationException


|  |  |  |
| --- | --- | --- |
| **Overview** \| **Package** \| **Class** \| **Use** \| **Tree** \| **Deprecated** \| **Index** \| **Help** | |  |
| PREV   NEXT | **FRAMES**    **NO FRAMES**     **All Classes** |  |


---


## **Uses of Class sbrt.shell.ApplicationException**

| Packages that use ApplicationException | |
| --- | --- |
| **sbrt.shell** | Provides classes used by shell-level software. |
| **sbrt.shell.mng.algebra** | Provides classes for creating algebra process managers. |
| **sbrt.shell.mng.fba** | Provides classes and interfaces for creating Flux Balance Analysis process managers. |
| **sbrt.shell.mng.geom** | Provides classes and for creating geometry process managers. |
| **sbrt.shell.mng.stat** | Provides classes and interfaces for creating statistical process managers. |
| **sbrt.shell.text** | Provides classes and interfaces for defining formats for basic objects. |

Uses of ApplicationException in sbrt.shell

| Methods in sbrt.shell that return ApplicationException | |
| --- | --- |
| `static ApplicationException` | `ProgramSolverFactory.interpret(ProgramSolverException e)`             Interprets a program solver exception and returns a corresponding application exception. |

Uses of ApplicationException in sbrt.shell.mng.algebra

| Methods in sbrt.shell.mng.algebra that return ApplicationException | |
| --- | --- |
| `static ApplicationException` | `LinearSolverManager.interpret(ExternalSoftwareException e)`             Interprets the provided external software exception. |

Uses of ApplicationException in sbrt.shell.mng.fba

| Methods in sbrt.shell.mng.fba that return ApplicationException | |
| --- | --- |
| `static ApplicationException` | `FbaOptManager.interpret(ConstraintViolationException e, FbaProcess<?> experiment)`             Returns an `ApplicationException` whose message describes the error that ocurred. |
| `static ApplicationException` | `CdHarFluxVectorManager.interpret(DimensionException e)`             Returns an `ApplicationException` whose message describes the error that ocurred. |
| `static ApplicationException` | `CdHarFluxVectorManager.interpret(ExceededLimitException e)`             Returns an `ApplicationException` whose message describes the error that ocurred. |
| `static ApplicationException` | `FbaOptManager.interpret(FbaOptException e, FbaProcess<?> experiment)`             Returns an `ApplicationException` whose message describes the error that ocurred. |
| `static ApplicationException` | `MsFluxIntervalManager.interpret(FluxRangeException e, FbaProcess experiment)`             Returns an `ApplicationException` whose message describes the error that ocurred. |
| `static ApplicationException` | `SimpleFluxIntervalManager.interpret(IllegalBoundsException e)`             Returns an `ApplicationException` whose message describes the error that ocurred. |
| `static ApplicationException` | `CdHarFluxVectorManager.interpret(InfeasibleSolutionException e)`             Returns an `ApplicationException` whose message describes the error that ocurred. |
| `static ApplicationException` | `CdHarFluxVectorManager.interpret(InvalidFluxVectorException e)`             Returns an `ApplicationException` whose message describes the error that ocurred. |
| `static ApplicationException` | `FbaOptManager.interpret(ProgramSolverException e)`             Returns an `ApplicationException` whose message describes the error that ocurred. |
| `static ApplicationException` | `CdHarFluxVectorManager.interpret(java.lang.RuntimeException e)`             Attempts to interpret the provided RuntimeException. |
| `static ApplicationException` | `FbaOptManager.interpret(java.lang.RuntimeException e, FbaProcess<?> experiment)`             Returns an `ApplicationException` whose message describes the error that ocurred. |
| `static ApplicationException` | `CdHarFluxVectorManager.interpret(UnboundedVariableException e)`             Returns an `ApplicationException` whose message describes the error that ocurred. |

Uses of ApplicationException in sbrt.shell.mng.geom

| Methods in sbrt.shell.mng.geom that return ApplicationException | |
| --- | --- |
| `static ApplicationException` | `CdHarManager.interpret(ExceededLimitException e)`             Returns an `ApplicationException` whose message describes the error that ocurred. |
| `static ApplicationException` | `CdHarManager.interpret(UnboundedVariableException e)`             Returns an `ApplicationException` whose message describes the error that ocurred. |

Uses of ApplicationException in sbrt.shell.mng.stat

| Methods in sbrt.shell.mng.stat that return ApplicationException | |
| --- | --- |
| `static ApplicationException` | `CorrelationManager.interpret(JriException e)`             Wraps the provided JRI exception as an application exception. |

Uses of ApplicationException in sbrt.shell.text

| Subclasses of ApplicationException in sbrt.shell.text | |
| --- | --- |
| `class` | `FormatException`             This exception type is used to indicate a formatting error has ocurred. |

---


|  |  |  |
| --- | --- | --- |
| **Overview** \| **Package** \| **Class** \| **Use** \| **Tree** \| **Deprecated** \| **Index** \| **Help** | |  |
| PREV   NEXT | **FRAMES**    **NO FRAMES**     **All Classes** |  |


---
